## [Peer Review File · Nature Communications]

Reviewers' comments:

Reviewer #1 (Remarks to the Author):

The manuscript by Brandau and colleagues reports on the importance of endosomal TLR deficiency for the induction of tumor rejection. Surprisingly, in contrast to wild type mice that were permissive to MOPC tumor growth, triple KO mice (TLR3, 7 and 9) totally rejected tumor cells within three weeks. Using bone marrow chimeras, the authors concluded that tumor rejection required both hematopoietic and non-hematopoietic cells. In this model of triple KO mice, tumor rejection was accompanied by increased size of vessels, tumor lymphocyte infiltrates, and dependence on both CD4 and CD8 T cells. The authors conclude that the endogenous anti-tumor immune response is negatively regulated by endosomal TLRs.

The paper is well written and the observations are original and surprising. However, their interpretation raises some important concerns. The presentation of the data often prevents drawing conclusions with confidence. Therefore, the manuscript does not reach the standards required for publication in Nature Communications.

Main concerns:

1. The specificity of the effects is not established. The fact that both hematopoietic and non-hematopoietic cells contribute to the observed phenotype calls for further experiments to decipher their respective contribution. Surprisingly, single and double KO mice exhibit much larger tumors than wild type mice (compare Fig S1 and Fig1a), suggesting that each of the 3 TLRs is required to limit or control tumor growth in wild type mice (this important point goes against the main conclusion of the study and is not commented on by the authors.). Indeed, CD4 T cell depletion - and to some extent CD8 depletion - accelerated tumor growth in wild type mice, indicating that T cells can partially limit tumor growth in wild type mice. Tumor rejection in triple KO mice was equally affected by CD4 cell and CD8 T cell depletion, but may reflect better accessibility of T cells to the tumor. Indeed the authors report that the diameter of the vessels within the tumors was increased in the triple KO mice. Therefore, increase in CD4 and CD8 T cell numbers observed in tumor infiltrates may be due to the increased vessel size.

It remains difficult to integrate all the results together. The triple KO mice may have some indirect effects on tissue homeostasis, stroma cell behavior, etc. restricting the permissiveness of the micro-environment allowing for tumor growth and/or favoring the arrival of immune cells.

2. To be convincing, presentation of the data would greatly benefit from plotting individual mouse raw data rather than means (one panel per type of mice), see Fig 1a, Fig 2a, Fig3a and g, Fig 4d, Fig 5a. Moreover, when individual mouse data are plotted, they reveal a very high heterogeneity within each group of mice (see for instance fig 2b and S1), further supporting the need for the reader to have access to raw data.

3. Comparisons of figures 1a and S1 reveals that tumor volumes are smaller in wt mice than in single and double KO. Given the heterogeneity observed in the results in Fig S1, fig 1a should be presented the same way. Here it looks like all triple KO mice exhibited the very same behavior, which seems highly unlikely.

4. The challenge experiments presented do not really fit with the idea of an immune response against the tumor since the secondary response appears slower than the primary one.

5. The differences in the inflammatory responses between wt and triple KO mice presented in Fig 4 c to e appear rather weak. Fig 4d should show individual mice rather than pools of 3 mice and should be compared to wild type mice.

Minor points:

The referee suggests that a control with the UNC93B1 KO mice or with the so-called 3d mice (from B Butler's lab) could be provided to establish in a simple manner the contribution of all the UNC93B-dependent TLRs, i.e. the nucleic acid sensor TLRs to tumor rejection.

Figure S1: Wild type control mice should be presented.

Figure 3E. The authors exclude that the endothelial cells die in the Tlr3/7/9-/- mice only by measuring caspase3 (one of the marker of apoptosis) expression by IF. This could be confirmed with another readout

Line 239: "TLRs have evolved as important sensors..." should be changed in "TLRs have evolved important sensors".

Reviewer #2 (Remarks to the Author):

Overall, this is a surprising study related to the role of TLRs in tumor growth control. There are strong animal models and the use of several different tumor models that show the same response strengthens the significance of the findings. Chimera studies show dependency on both hematopoietic and non-hematopoietic cells on the phenomena observed. The use of a rechallenge experiments confirm long-lived immunity.

Since many studies are trying to bolster anti-tumor immunity using agonists against these TLRs, this report may provide rationale to block these receptors instead.

There are several concerns which should be addressed:

Orthotopic readout- Since the authors are injecting orthotopically in tongue, how do they know that the weight loss observed is not simply due to impedance of food intake by the tongue (i.e., an inability to eat normally.)

Immune Phenotype: Additional characterization of activation and functionality of CD4 and CD8 T cells is needed. CD4 positive cells could be TReg and CD4 and CD8 could be non-functional or exhausted. There should be a stain for IFN γ or other functional markers.

How are T cells being activated? Is there an impact on DCs? i.e., on expression of MHC-I/II, CD80/86 ?

Regarding the mechanism by which T cells are getting into tumor several questions arise:

Is the increase in T cells due to their proliferation at the tumor site rather than a change in trafficking?

Is this a T cell intrinsic effect? (i.e., E/P selectin, LFA-1)

- Endothelial cell effect? (i.e., ICAM-1 expression)

Are T cells simply "leaking" into tumor paranchyma? Please clarify.

- What is known about the animal model?

- i.e., BM make-up, circulating blood composition, etc.
- CBC?
- has it been used in any other models (i.e., infection) to see if response is also robust?

When do blood vessels start changing and could this explain tumor regression? Tumors consistently regress at or before 10 days, but the authors do not look at vessel density or perfusion or diameter. Do the blood vessels change before BEFORE regression begins?

Reviewer #1 (Remarks to the Author):

The manuscript by Brandau and colleagues reports on the importance of endosomal TLR deficiency for the induction of tumor rejection. Surprisingly, in contrast to wild type mice that were permissive to MOPC tumor growth, triple KO mice (TLR3, 7 and 9) totally rejected tumor cells within three weeks. Using bone marrow chimeras, the authors concluded that tumor rejection required both hematopoietic and non-hematopoietic cells. In this model of triple KO mice, tumor rejection was accompanied by increased size of vessels, tumor lymphocyte infiltrates, and dependence on both CD4 and CD8 T cells. The authors conclude that the endogenous anti-tumor immune response is negatively regulated by endosomal TLRs.

The paper is well written and the observations are original and surprising. However, their interpretation raises some important concerns. The presentation of the data often prevents drawing conclusions with confidence.

Response: We thank the reviewer for his positive assessment of our original findings. We have performed additional experiments, which address the concerns of both reviewers. We have also optimized data presentation according to the reviewer's suggestion. We are convinced that these changes substantially improved the manuscript and thank both reviewers for their suggestions and comments.

Main concerns:

1. The specificity of the effects is not established. The fact that both hematopoietic and non-hematopoietic cells contribute to the observed phenotype calls for further experiments to decipher their respective contribution.

Response: Indeed, the contribution of both compartments suggests a complex mechanism and phenotype involved in tumor rejection. As outlined in this point-by-point-response we have added multiple experiments providing more information on the contribution of immune and non-immune cells to tumor rejection.

Surprisingly, single and double KO mice exhibit much larger tumors than wild type mice (compare Fig S1 and Fig1a), suggesting that each of the 3 TLRs is required to limit or control tumor growth in wild type mice (this important point goes against the main conclusion of the study and is not commented on by the authors.).

Response: As suggested below (comment on figure S1) we have added the WT control data to suppl figure S1 and now show these controls and all double-ko combinations.

As this reviewer knows, depending on the cell line used, tumor growth curves may vary between mice and between cell culture batches of tumor cells used. Fig S1 now shows individual mice from different genotypes all performed with one batch of tumor cells in a single experiment in order to allow direct comparison of tumor growth curves within one experiment. In essence, these data re-emphasize robust tumor growth in WT and all double-ko combinations, in contrast to rejection in triple-ko. While a slightly reduced tumor growth in TLR3/9 double ko seems to occur, we do not think that our data justify conclusions on substantial differences in tumor growth between the WT and the double KO genotypes. Instead we would like to re-emphasize and focus the work on the dramatic rejection phenotype observed in triple KO mice.

Indeed, CD4 T cell depletion - and to some extent CD8 depletion - accelerated tumor growth in wild type mice, indicating that T cells can partially limit tumor growth in wild type mice.

Response: Yes, this is correct and shown in figure 6a. We also mention this fact on page 9, line 239. Nevertheless, we focus this manuscript on the by far greater effect of T cell depletion in TLR triple ko mice, also shown in figure 6. In the ko mice, depletion of T cells converts tumor growth from rejection into a growth curve very similar to WT mice.

Tumor rejection in triple KO mice was equally affected by CD4 cell and CD8 T cell depletion, but may reflect better accessibility of T cells to the tumor. Indeed the authors report that the diameter of the vessels within the tumors was

increased in the triple KO mice. Therefore, increase in CD4 and CD8 T cell numbers observed in tumor infiltrates may be due to the increased vessel size.

Response: We agree with the reviewer that increased vessel size may alter accessibility of the tumor for T cell influx. It is, however, if at all, very difficult to experimentally address a causal link between vessel size and T cell influx. Along this line, reviewer #2 brought up a potentially increased leakiness of vessels in TLR KO mice. We have performed additional experiments, which clearly demonstrate that tumor-infiltrating T cells in TLRko mice are strongly activated and positive for Granzyme B and CD107a (new figure 5). In addition, both CD4 and CD8 TIL show increased cytokine production. Thus, we conclude that not only T cell numbers but also the state of T cell activation differs substantially between WT and TLRko. This also shows that a T cell-intrinsic effect is likely to contribute to tumor rejection. In addition, we have employed fluorescently labelled Dextran (see below, reviewer #2) to test the leakiness of vessels in WT and TLR KO. Experiments revealed no substantial differences between both mouse strains.

It remains difficult to integrate all the results together. The triple KO mice may have some indirect effects on tissue homeostasis, stroma cell behavior, etc. restricting the permissiveness of the micro-environment allowing for tumor growth and/or favoring the arrival of immune cells.

Response: We completely agree with the reviewer that multiple highly interesting mechanisms and scenarios may be involved and even synergize in this model of tumor regression. While it is impossible to experimentally address all these evenly complex potential mechanisms, in the revised version, we have performed several experiments based on concrete suggestions made by reviewer #2, which address the vessel composition and the functional T cell phenotyping in tumors from KO vs WT mice (see below, comments from rev #2).

In addition we discuss now in more detail the above mentioned possibilities explaining increased T cell infiltrates in tumors (substantially amended discussion, page 12).

In sum our data are consistent with the following scenario: between day 6 and day 10 T cell proliferation is induced in tumor draining LN (new figure 5a). The combined activity of tumor-resident stromal cells and immune cells drives T cell recruitment into the tumor microenvironment (figure 2, figure S3b). While vessel leakiness did not differ between control WT and KO regressor mice (exp performed during revision, data not shown in Ms), signs of vessel normalization occur in TLRKO mice (figure 3f, g). At the tumor site recruited T cells activate a broad effector response, which includes cytokine production and cytolytic activity (new figure 5b, c and figure S3d). Concomitantly, the induction of Treg, seen in WT, is inhibited in tumors of TLRKO mice (figure S3c).

2. To be convincing, presentation of the data would greatly benefit from plotting individual mouse raw data rather than means (one panel per type of mice), see Fig 1a, Fig 2a, Fig3a and g, Fig 4d, Fig 5a. Moreover, when individual mouse data are plotted, they reveal a very high heterogeneity within each group of mice (see for instance fig 2b and S1), further supporting the need for the reader to have access to raw data.

Response: We now show individual mice in Fig S1, Fig S2, Fig S3, Fig 1c-e, Fig 2, Fig 3b, c, g, Fig 3g, and most panels of figure 4. For clarity the heterogeneity in Fig 1a and Fig 4d is shown by depicting the SEM of the data. I hope the reviewer agrees that for the sake of clarity showing the mean is required and beneficial in some selected figure panels.

Data from figure 6a are additionally shown as individual mouse data in a figure at the bottom of this cover letter (panel b, additional information, see below).

3. Comparisons of figures 1a and S1 reveals that tumor volumes are smaller in wt mice than in single and double KO. Given the heterogeneity observed in the results in Fig S1, fig 1a should be presented the same way.

Response: Please see above for responses to these two comments.

Here it looks like all triple KO mice exhibited the very same behavior, which seems highly unlikely.

Response: Certainly, the transplanted tumors show an expected degree of heterogeneity in their growth curves. During the project we have accumulated data from a substantial number of WT and triple KO mice. While in WT mice tumors

continuously grow, more than 95% of TLR triple KO mice show INITIAL tumor growth followed by tumor shrinkage and REJECTION within 3-4 weeks. Please see tumor take rate (=palpable tumor) in Fig 1a and Fig S2. Thus the difference between WT and TLRKO is very significant. Because of this very reproducible growth curves the standard deviation is quite small in TLRKO mice. To meet the request of this reviewer data from individual mice for WT versus triple KO are additionally shown in Fig S1b.

4. The challenge experiments presented do not really fit with the idea of an immune response against the tumor since the secondary response appears slower than the primary one.

Response: This seems to be a misunderstanding and apparently figure 6c was not explained well enough. We have modified the figure and now indicate the time point of the second tumor injection (challenge for regressor TLRKO mice). At the same time point the two control groups (naive tumor free TLRKO and naive tumor free WT) also received the tumor injection. We have also improved the description of the experiment in the results section (page 9).

5. The differences in the inflammatory responses between wt and triple KO mice presented in Fig 4 c to e appear rather weak. Fig 4d should show individual mice rather than pools of 3 mice and should be compared to wild type mice.

Response: We agree with the reviewer that differences in cellular influx (Fig 4a-b) are stronger compared to inflammatory cytokines (fig 4c-e). Nevertheless clear statistically significant differences were measured. Quantitative PCR for figure 4d was repeated and now shows individually measured mice including mean and SEM. Figure 4c,e compare WT and triple ko, while Fig 4d displays the time course of cytokine expression in TLRKO. Since exact and absolute quantification of cytokine protein levels in tissues in situ is almost impossible to do, we believe that the ELISA data from tumor explants in figure 4e are quite informative.

Minor points:

The referee suggests that a control with the UNC93B1 KO mice or with the so-called 3d mice (from B Butler's lab) could be provided to establish in a simple manner the contribution of all the UNC93B-dependent TLRs, i.e. the nucleic acid sensor TLRs to tumor rejection.

Response: Unfortunately, we were not in the position to use the suggested mouse strain. To accommodate this comment we have used MyD88/TRIF-/- mice.

[Redacted]

Figure S1: Wild type control mice should be presented.

Response: Were included.

Figure 3E. The authors exclude that the endothelial cells die in the Tlr3/7/9-/- mice only by measuring caspase3 (one of the marker of apoptosis) expression by IF. This could be confirmed with another readout

Response: Caspase 3 immunofluorescence is a very common read-out to determine cell type-specific apoptosis in tissue sections. We tried to establish alternative staining protocols to co-stain for CD31 and apoptotic markers, but were unfortunately not successful. In an alternative approach we performed flow cytometry viability dye measurements on CD31+ cells in tumor suspensions. These measurements also revealed no difference between WT and TLRKO. However, most likely, this method also marks cells as dead, which are harmed during technical preparation of the tissue. Thus, it is not a "true" quantification of cell death in vivo / in situ. As a consequence of these technical challenges, we have softened the statement on apoptosis in the text.

Line 239: "TLRs have evolved as important sensors..." should be changed in "TLRs have evolved important sensors".

Response: Sentence has been changed.

Reviewer #2 (Remarks to the Author):

Overall, this is a surprising study related to the role of TLRs in tumor growth control. There are strong animal models and the use of several different tumor models that show the same response strengthens the significance of the findings. Chimera studies show dependency on both hematopoietic and non-hematopoietic cells on the phenomena observed. The use of a rechallenged experiment confirms long-lived immunity.

Since many studies are trying to bolster anti-tumor immunity using agonists against these TLRs, this report may provide rationale to block these receptors instead.

There are several concerns which should be addressed:

Orthotopic readout- Since the authors are injecting orthotopically in tongue, how do they know that the weight loss observed is not simply due to impedence of food intake by the tongue (i.e., an inability to eat normally.)

RESPONSE: As a matter of fact, and similar to the clinical situation in humans, the tumor growth impedes feeding. This causes initial weight loss in ko mice. Post day 10 mice gain weight again because of tumor regression and normalization of food intake. In WT mice, progressive tumor growth leads to substantial weight loss, which requires sacrifice of mice. Monitoring of weight, rather than tumor size, is the primary read-out in this tumor model. This is caused by local animal welfare regulations, which do not allow us to continue experiments beyond 20% body weight loss. At the end of the experiment tumor growth as well as rejection was confirmed by autopsy of mice. Thus, these data confirm the tumor rejection phenotype and show that the rejection is not limited to the s.c. site.

Immune Phenotype: Additional characterization of activation and functionality of CD4 and CD8 T cells is needed. CD4 positive cells could be TReg and CD4 and CD8 could be non-functional or exhausted. There should be a stain for IFN γ or other functional markers.

Response: We fully agree with the reviewer that the study would benefit from additional characterization of CD4/CD8 activation, functionality and phenotype. We have performed the following experiments to address this point:

As suggested we stained for IFN- γ . Results are presented in figure 5c. The data show that CD4 and CD8 isolated from tumors of TLRko produce more IFN- γ (and TNF- α) than their counterparts isolated from WT mice.

As suggested we also looked into Treg. Data are presented in Fig S3c. Results show that the percentage of tumor-infiltrating Treg is increased in WT over TLRko.

We also tested several markers of T cell exhaustion or dysfunctionality such as PD-1, LAG3 and TIM3. However, no significant difference in the expression of these markers was found in T cells isolated from WT versus TLRko.

As further detailed below, we also found increased numbers of CD8 effector cells (GrzB+/perforin+/CD107a+) in tumors from TLRKO mice.

In conjunction these data show that tumor in WT mice are infiltrated by increased numbers of Treg, while tumors in TLRKO display a high number of effector T cells. These differences are not present until day 6 (tumor growth phase), but become apparent by day 10 (rejection phase). These findings underscore the importance of T cell activation and T cell-mediated anti-tumor immunity for tumor regression in TLRKO mice.

How are T cells being activated? Is there an impact on DCs? i.e., on expression of MHC-I/II, CD80/86 ?

Response: Similar to this reviewer we also speculated that DC could play an important role during induction of tumor regression. Already in the original work and later in the revision phase we performed several experiments into this direction. We used both bone-marrow derived DC and primary DC isolated from LN and tumor. We tested intrinsic DC activation, maturation status and response to stimulation and incorporated all markers suggested by this reviewer.

However, we could not detect significant differences between DC from WT and TLRKO. We carefully analyzed CD80 and CD86 expression as suggested. Both markers were equally expressed between tumor DC from both mouse strains. On LN DC a slight, but statistically not significant, increase in both markers was present in TLRKO on day 10 (not included in Ms).

Regarding the mechanism by which T cells are getting into tumor several questions arise:

Response: We fully agree with this reviewer that multiple mechanisms could be involved and cause the striking difference in CD4/CD8 TIL. We addressed all suggested effects experimentally.

Is the increase in T cells due to their proliferation at the tumor site rather than a change in trafficking?

Response: We measured proliferation of T cells in LN and tumor by Ki67 IHC. We found that the proliferation of CD4 and CD8 cells is increased in LN of TLRKO over WT. Data are shown in figure 5a. No difference in the proliferation of tumor infiltrating T cells was found. Data were confirmed by using BrdU in vivo labelling as a second method (not shown). These data suggest that the increase in T cells is not due to increased intratumoral proliferative activity, but rather increased trafficking.

Is this a T cell intrinsic effect? (i.e., E/P selectin, LFA-1)

Response: We have investigated several adhesion molecules and effector molecules on TIL and blood CD4 and CD8. We could not detect robust and consistent differences in expression of selectins/adhesion molecules between T cells from WT and TLRKO. Instead we observed substantial differences in the expression of CD154, CD107a, Perforin, GrzB and these data are shown in figure 5b and figure S3d. Our results show that tumors from TLRKO, but not WT, are infiltrated by CD4 and CD8 cells, which show an activated effector cell phenotype. This difference was measured at day 10 (rejection phase), but was not yet present at day 6 of tumor growth (new figure 5).

- Endothelial cell effect? (i.e., ICAM-1 expression)

Response: We measured the expression of CD54 on CD45-/CD31+ cells from tumor suspensions by flow cytometry and analyzed CD54 expression on vessels by IHC. The data suggested an increase of CD54 on vessels between day 6 and day 10 of tumor growth in both mouse strains. This increase was less pronounced in TLRKO compared with WT and, in our view, is unlikely to explain the differences in the tumor microenvironment of WT and TLRKO mice.

Are T cells simply "leaking" into tumor parenchyma? Please clarify.

Response: We have performed a vessel perfusion experiment with fluorescently tagged Dextran to address this point. Our data showed no difference between WT controls and tumor regressor mice using this approach, which makes a passive leaking of T cells into the tumor unlikely. This assumption is further supported by the fact that no signs of vessel activation could be observed in TLRKO mice. Vessel normalization (pericyte coverage, day 14) in TLRKO mice occurred slightly after the induction of T cell recruitment. Lastly, we report a very substantial difference in T cell activation between WT and KO, which suggests that T cell activation at least seems to contribute to the inflamed tumor microenvironment phenotype observed in this study.

All in all our data suggest the following scenario: Between day 6 and day 10 T cell proliferation is induced in tumor draining LN. An enhanced inflammatory response of tumor-resident stromal cells drives T cell recruitment into the tumor microenvironment. At the tumor site recruited T cells activate a broad effector response, which includes cytokine production and cytolytic activity. This T cell recruitment and activation is accompanied by selected and specific changes in the tumor vasculature. While vessel activation and leakiness remain unchanged in the KO regressor mice, microvessel

density decreases and tumors present with fewer and larger vessels, which additionally acquire pericyte/desmin coverage as late as day 14.

- What is known about the animal model?
- i.e., BM make-up, circulating blood composition, etc.
- has it been used in any other models (i.e., infection) to see if response is also robust?

Response: To the best of our knowledge the TLR3/7/9 KO combination has not been used in infection models and we are not aware of systematic analyses of BM and blood leukocyte compositions in these mice. Only combinations of two receptor deficiencies (TLR3/7, TLR7/9) have been tested for example in HIV/AIDS models. The only study using TLR3/7/9 KO mice utilized aged mice (age of 6 month to 15 month) and describes the reactivation of endogenous retroviruses in these animals. In our study we used young mice (6-16 weeks) and performed comparative analysis of blood and BM with a focus on T cells and myeloid cells in order to be able to provide some information on this comment. Composition of BM and blood was similar for both mice strains for CD4 and CD8 cells as well as subsets of CD11b+ myeloid cells (myeloid precursors, monocytes/macrophages, granulocytes, dendritic cells). T cells were in the normal range (around 10% for CD8 and around 20% for CD4) in both mouse strains. Some data are shown in figure S3a.

When do blood vessels start changing and could this explain tumor regression? Tumors consistently regress at or before 10 days, but the authors do not look at vessel density or perfusion or diameter. Do the blood vessels change before BEFORE regression begins?

Response: We thank the reviewer for highlighting this point and for allowing us to make this point clearer in the manuscript. Our data now clearly show that vessels do NOT CHANGE BEFORE regression begins. Figure 3 has been modified accordingly and a clear time-dependent development of differences in vessels is now shown in figure 3b, figure 3c, and figure 3g.

General Response to Reviewer #2:

We thank Rev #2 for his careful assessment of our study and for his detailed and concrete suggestions on how to improve the manuscript. Based on the additional experiments and data we now have a clearer picture on the effects which contribute or most likely do NOT contribute to the induction of T cell-mediated immunity between day 6 and day 10 in this tumor model of endosomal TLR-deficiency on host cells.

REVIEWERS' COMMENTS:

Reviewer #1 (Remarks to the Author):

The manuscript has been improved. The precise mechanism(s) underlying the efficient immune control of tumor growth observed in TLR3/7/9 deficient mice remains elusive. However the data are solid, surprising, compelling and important for the field and thus deserve to be published in Nature Communications.

Reviewer #2 (Remarks to the Author):

I believe the authors have satisfactorily addressed most of my concerns from the first review. The new experiments are useful and help confirm the original data. The authors have also commented upon various potential mechanisms in the Discussion.

One minor suggestion is to include potential relationships now emerging between microbiome and endogenous anti-tumor immune control as a tumor model specific variable, in the Discussion.

Point by point reply:

Reviewers comment:

Reviewer #2 (Remarks to the Author):

I believe the authors have satisfactorily addressed most of my concerns from the first review. The new experiments are useful and help confirm the original data. The authors have also commented upon various potential mechanisms in the Discussion.

One minor suggestion is to include potential relationships now emerging between microbiome and endogenous anti-tumor immune control as a tumor model specific variable, in the Discussion.

Response:

On the first page of the discussion we have added a paragraph to briefly discuss this emerging topic. We cited three key papers in this paragraph.