# Supplementary material for: Combined toll-like receptor 3/7/9 deficiency on host cells results in T-cell-dependent control of tumour growth
Source: Nat Commun. 2017 Mar 16;8:14600. doi: 10.1038/ncomms14600 (PMC5356072; doi:10.1038/ncomms14600)
Supplement: Supplementary Information — Supplementary Figures and Supplementary Table [file ncomms14600-s1.pdf]

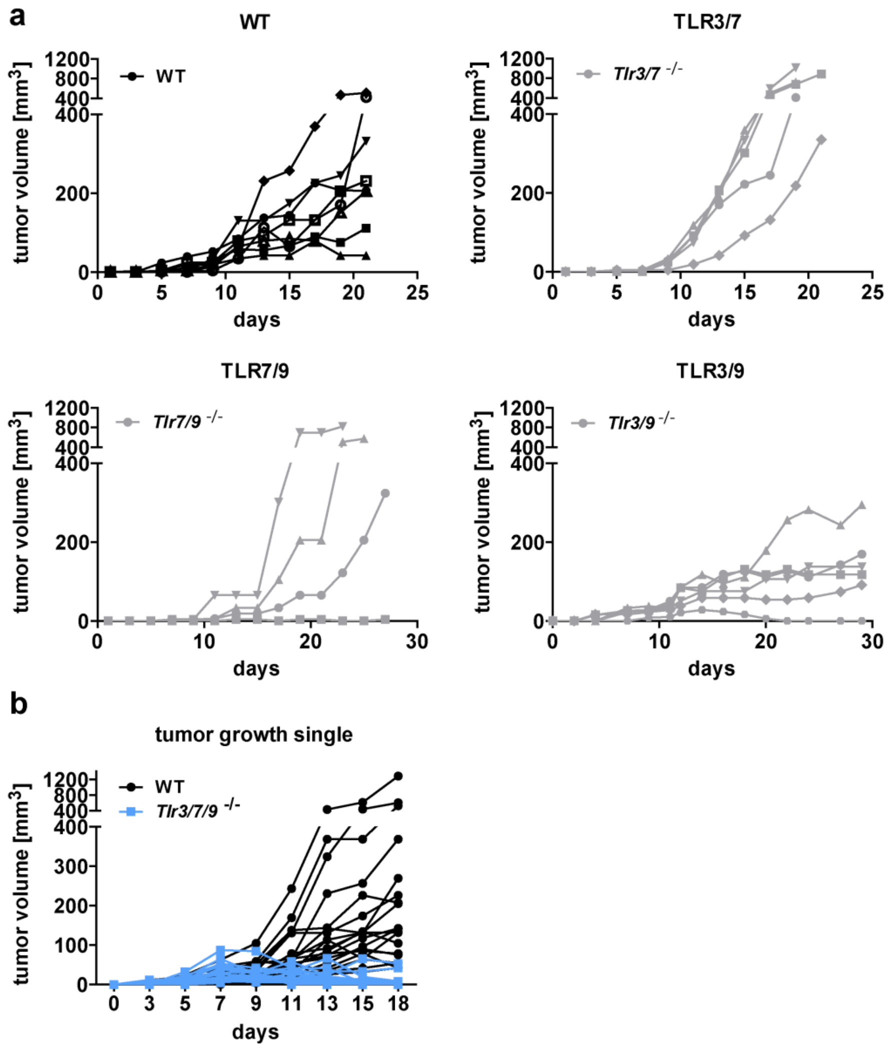

Supplementary Figure 1. Tumor growth in WT double and triple *Tlr*-KO mice.

(a) Tumor growth of MOPC tumors was additionally investigated in *Tlr* double KO mice. 0.5 x 10^6 MOPC tumor cells were injected s.c. into seven- to fifteen-weeks old C57BL/6 WT (n=8), *Tlr3/7*$^{-/-}$ (n=5), *Tlr7/9*$^{-/-}$ (n=4) and *Tlr3/9*$^{-/-}$ mice (n=6) and tumor growth was measured every other day. Data for individual mice are shown.**(b)** Single MOPC tumor growth curves in eight- to sixteen-weeks old C57BL/6 WT (n=19) and *Tlr3/7/9*$^{-/-}$ mice (n=24).

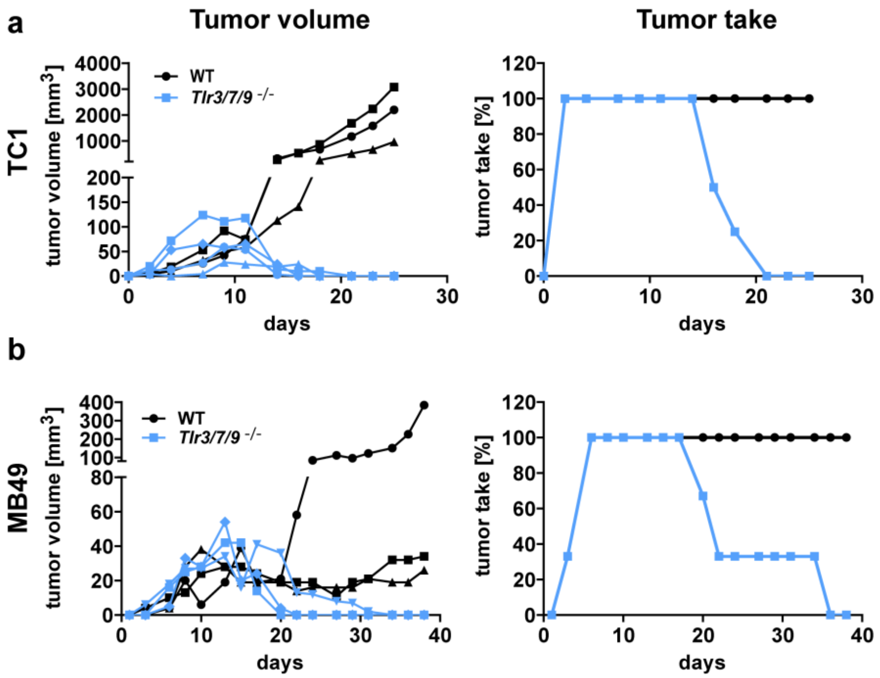

Supplementary Figure 2. ***Tlr3*/7/9$^{-/-}$ mice reject also other tumor cell types.**

Tumor growth of additional tumor cell lines was investigated in seven- to sixteen-weeks old C57BL/6 WT and *Tlr3/7/9*$^{-/-}$ mice. 2 x 10^6 TC1 lung tumor cells **(a)** or 0.5 x 10^6 MB49 bladder tumor cells **(b)** were injected s.c. into WT (n=3) and *Tlr3/7/9*$^{-/-}$ mice (n=3-4). Tumor growth was measured every other day. Left panels depict tumor volume curves for individual mice and right panels depict the percentage of mice with palpable tumors (= take rate) within the respective experimental groups.

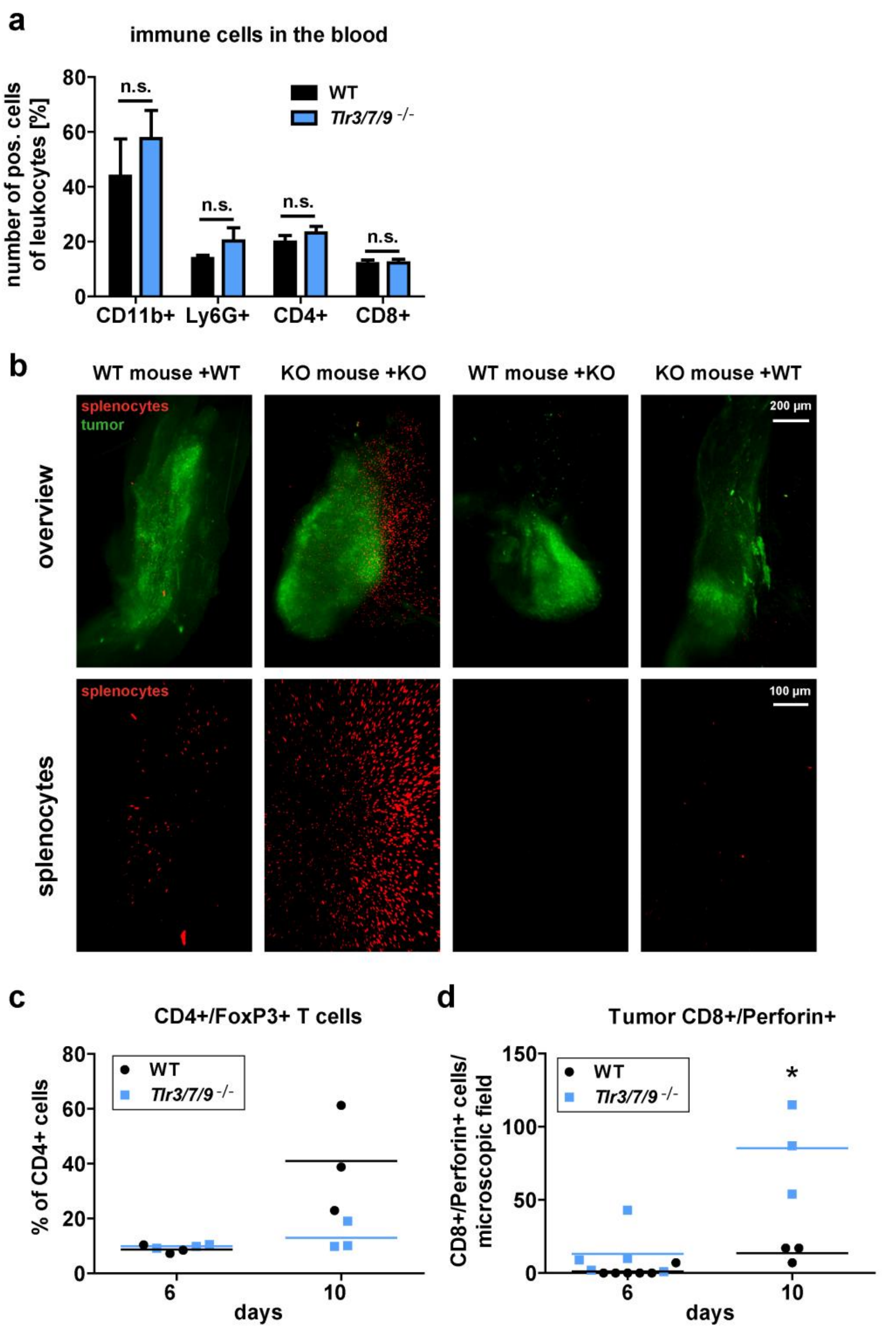

Supplementary Figure 3. Supplementary immune cells analysis.

(a) Flow cytometry of peripheral blood of day 10 tumor bearing eight- to sixteen-weeks old C57BL/6 WT and *Tlr3/7/9*$^{-/-}$ mice using αCD11b, αLy6G, αCD4 and αCD8 antibodies. Number of cells was calculated in percent to total number of leukocytes. Mean of minimum of three mice + S.E.M is shown.P-value calculated by t-test. **(b)** 1 x 10^6 green fluorescent MOPC^{-eGFP} were injected into recipient mice. Splenocytes of ten- to twelve-weeks old C57BL/6 WT and *Tlr3/7/9*$^{-/-}$ tumor bearing donor mice were isolated, labeled with CellTracker™ Deep Red Dye and

adoptively transferred into recipient mice at a dose of 1 x 10^7 labeled splenocytes per mouse. One day later tumors were dissected and chemically cleared. Infiltration of transferred splenocytes was investigated by generating 3D images using the light sheet technology and Imaris software. One out of two tumors per group is shown. **(c, d)** Quantification of tumor infiltrating regulatory T cells (c) and perforin-positive CD8 cells (d) in six- to twelve-weeks old C57BL/6 WT and *Tlr3/7/9*$^{-/-}$ mice at day 6 and day 10 by flow cytometry using αCD4 and αFoxP3 antibodies (c) or by immunohistochemistry using two-color immunofluorescence (d). Number of mice was a minimum of 3 and horizontal line shows the mean value. Results were considered significant at *$p \leq 0.05$ (t test).

Supplementary Table 1. PCR primer sequences.

Gene	Direction	Primer sequence
IL-6	forward	GAGTGGCTAAGGACCAAGACC
	reverse	AACGCACTAGGTTTGCCGA
IL-8	forward	CCCCCATCCCCATTTCTTGAT
	reverse	AGCCCATAGTGGAGTGGGATA
IL-24	forward	ACCAGCGGTTTTTGCTGTTC
	reverse	TCTGCATCCAGGTCAGGAGA
IFN-γ	forward	ATTAGCCAAGACTGTGATTGCG
	reverse	CTGAGTTCAGTCAGCCGCTT

Cytokine gene expression in tumor tissues was quantitated by qRT-PCR using the indicated primer pairs.
